# Characteristics of non-fatal self-poisoning in Sri Lanka: a systematic review

**DOI:** 10.1186/1471-2458-13-331

**Published:** 2013-04-10

**Authors:** Thilini Rajapakse, Kathleen Margaret Griffiths, Helen Christensen

**Affiliations:** 1 Department of Psychiatry, Faculty of Medicine, University of Peradeniya, Peradeniya, Sri Lanka; 2 Centre for Mental Health Research, Building 63, The Australian National University, Canberra, ACT 0200, Australia; 3 Black Dog Institute, University of New South Wales, Hospital Road, Randwich, NSW 2031, Australia

## Abstract

**Background:**

The rate of non-fatal self-poisoning in Sri Lanka has increased in recent years, with associated morbidity and economic cost to the country. This review examines the published literature for the characteristics and factors associated with non-fatal self-poisoning in Sri Lanka.

**Methods:**

Electronic searches were conducted in Psychinfo, Proquest, Medline and Cochrane databases from inception to October 2011.

**Results:**

26 publications (representing 23 studies) were eligible to be included in the review. A majority of studies reported non-fatal self-poisoning to be more common among males, with a peak age range of 10–30 years. Pesticide ingestion was the most commonly used method of non-fatal self-poisoning. However three studies conducted within the last ten years, in urban areas of the country, reported non-fatal self-poisoning by medicinal overdose to be more common, and also reported non-fatal self-poisoning to be more common among females. Interpersonal conflict was the most commonly reported short-term stressor associated with self-poisoning. Alcohol misuse was reported among males who self-poisoned, and data regarding other psychiatric morbidity was limited.

**Conclusions:**

The findings indicate that pesticide ingestion is the commonest method of non-fatal self-poisoning in Sri Lanka, and it is more common among young males, similar to other Asian countries. However there appears to be an emerging pattern of increasing medicinal overdoses, paralleled by a gender shift towards increased female non-fatal self-poisoning in urban areas.

Many non-fatal self-poisoning attempts appear to occur in the context of acute interpersonal stress, with short premeditation, and associated with alcohol misuse in males. Similar to other Asian countries, strategies to reduce non-fatal self-poisoning in Sri Lanka require integrated intervention programs with several key aspects, including culturally appropriate interventions to develop interpersonal skills in young people, community based programs to reduce alcohol misuse, and screening for and specific management of those at high risk of repetition following an attempt of self-poisoning.

## Background

Sri Lanka has a high rate of suicide, which reached a peak in 1995, at 47 per 100,000 population [1,2]. Vijayakumar et al., in their review of suicide in developing countries reported that the average annual suicide rate for the 1990s in Sri Lanka was 21.6/100,00, a level that was high compared to rates in neighbouring India (9.7/100,000) and China (16.1/100,000) [3]. Pesticide poisoning, estimated to account for up to one-third of the world’s suicides [4], is the most common method of suicide in Sri Lanka, and in other South Asian countries [5-7]. Since 1995 the suicide rate in Sri Lanka has declined to 23 per 100,000 population (2006), a reduction that has been attributed to a drop in case fatality following reduction in toxicity of accessible pesticides [1]. Despite the drop in rate of completed suicide, the rates of non-fatal self-poisoning continues to be high [8,9]. The rate for non-fatal self-poisoning for males, as reported by a regional study in South Sri Lanka in 2002 was 330/100,000 [8] which is comparable to the highest average age standardized attempted suicide rates for males reported by the WHO/EURO para-suicide study of 314/100,000 [10]. Non-fatal self-poisoning is also associated with significant morbidity and economic cost to the country – for example, the cost of treating patients after self-poisoning in all of Sri Lanka in 2004 was estimated to be US$ 866,304 [11].

Over the past 40 years, several studies have investigated the incidence rates and factors associated with non-fatal self-poisoning in Sri Lanka [7,9,12]. The findings from these studies suggest that non-fatal self-poisoning is a phenomenon of young people and that it is commonly seen in both genders [7,9,12]. The studies also suggest that factors associated with non-fatal self-poisoning in Sri Lanka may differ from those which operate in Western countries. In particular, the repetition rates (i.e., the suicide reattempt rates) appear low, and there are lower reported rates of associated psychiatric morbidity [13,14]. However, the heterogeneity of these Sri Lankan studies makes overall conclusions difficult. For example, most studies have been conducted in different regions of the country, some based in the community, and others based on hospital admission data. The studies are of varying sample size, design and duration, and the nature of the poisoning also varies. For example some studies examined pesticide self-poisoning only, some examined all types of self-poisoning, and some did not differentiate between intentional and accidental self-poisoning. To date there has been no overall synthesis of available evidence regarding attempted self-poisoning in Sri Lanka.

The main aim of this systematic review is to examine relevant published literature, in order to describe the rates, socio-demographic characteristics, risk factors such as psychiatric morbidity and previous self-harm associated with non-fatal self-poisoning in Sri Lanka. Based on the findings of this review, we also discuss non-fatal self-poisoning in Sri Lanka in the context of non-fatal self-poisoning patterns internationally, with particular reference to cross national World Health Organization studies relevant to attempted suicide [13,15]. Finally, based on the findings of this review, we also aim to discuss potential interventions to reduce non-fatal self-poisoning rates in Sri Lanka – including both first attempts, and repetitions. It is anticipated that a review of the literature from Sri Lanka will suggest directions for the development of effective intervention programs for reducing non-fatal self-poisoning rates in this country. Strategies to reduce mortality due to medical causes following non-fatal self-poisoning is beyond the scope of this review, and hence is not discussed.

## Method

### Search strategy for identification of studies

**Figure 1:**
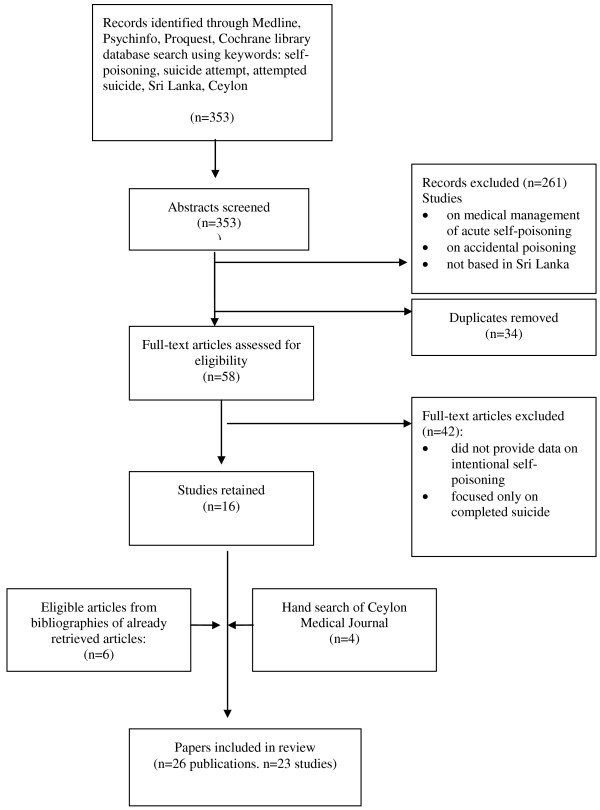
Flow diagram for literature search and review.

Electronic searches were conducted across bibliographic databases, namely Psychinfo, ProQuest Central, Medline and the Cochrane library from inception to 31^st^ October 2011. Eligible studies published in indexed journals in the English language were located using combinations of the following MeSH terms and keywords: self-poisoning, suicide attempt, attempted suicide, Sri Lanka, Ceylon (Figure 1). English is the medium used for scientific publication in Sri Lanka, and hence the search was conducted in English. The title and abstracts of all articles were perused initially, and those that clearly did not meet inclusion criteria were excluded. The full text of all remaining articles was retrieved and examined for eligibility to be included in the review, and the final decision to include an article was based on perusal of the full report. In addition, bibliographies of retrieved articles were also examined for relevant studies, and potentially relevant papers were then retrieved and reviewed using the above selection criteria. Print issues of the Ceylon Medical Journal were also hand-searched from it’s inception in 1954 onwards, for relevant papers, and print issues of The Journal of the Ceylon Branch of the British Medical Association, which is the forbear of the Ceylon Medical Journal was also hand-searched as far as possible (wherever print records were available, from it’s inception in 1887 onwards); however there was no search of the grey literature.

### Inclusion criteria

For the purposes of this review, the term ‘non-fatal self-poisoning’ is defined as self-poisoning attempts that were carried out intentionally, but with a non-fatal outcome. Thus, studies focusing on intentional, non-fatal self-poisoning in Sri Lanka, published in indexed journals, were eligible for inclusion in this review. Studies were also included if they reported on both accidental and intentional poisoning, provided that the intentional self-poisoning group could be clearly delineated for separate examination. Given the limited number of studies available, and to be as inclusive as possible, studies were included which exclusively considered survivors of intentional self-poisoning attempts, as well as those which considered participants who attempted intentional self-poisoning irrespective of outcome (i.e., which included those who survived as well as those who later died). No study was excluded on methodological grounds alone, and the search strategy included all types of study designs, including clinical trials, cross sectional descriptive studies and retrospective surveys.

The terminology used to describe suicidal behaviour and self-poisoning in Sri Lankan literature (as in the rest of the world) tends to vary considerably. Terms such as suicidal attempt, self-harm and self-poisoning are often used interchangeably and without accompanying definitions, and in many articles the degree of suicidal intent underlying the act is implied rather than described. In order to be as inclusive as possible, this review considered for inclusion all studies that focused on intentional, non-fatal self-poisoning in Sri Lanka, regardless of the terminology used to describe the behaviour. This included studies in which suicidal intent associated with self-poisoning was overtly stated, as well as studies in which suicidal intent was implied, based on the behaviour of the participants concerned.

### Exclusion criteria

Studies were excluded from the review if they were not conducted in Sri Lanka, if they were not written in English or if they exclusively reported data or outcomes which focused only on the medical management of acute self-poisoning. Since the aim of this review is to examine factors associated with non-fatal intentional self-poisoning in Sri Lanka, studies which focused solely on accidental poisoning, as well as those which focused solely on fatal self-poisoning or completed suicides in Sri Lanka were also excluded.

### Analysis strategy

A data extraction sheet was created, in order to code relevant information from each study included in the review, in a consistent manner. Outcomes of interest included study design, nature and size of sample, gender and age distribution, rates of self-poisoning, types of poison ingested and where available, degree of suicidal intent associated with the act, and factors associated with self-poisoning, such as triggers, psychiatric morbidity and alcohol use disorders. The method of assessment of outcomes such as psychiatric morbidity and alcohol use disorders was also noted for each study. Each included publication was scrutinized by one author (TR) initially, and relevant data was extracted and tabulated for each study, using the data extraction sheet. Subsequently the data extraction and articles were reviewed by all three authors, and areas of contention were discussed and decided upon jointly by all three authors.

## Results

As described above (Figure 1), after the initial database search, 353 abstracts were screened, of which 261 were excluded because the studies were either not based in Sri Lanka, focused only on accidental poisoning, or focused only on the medical management of acute self-poisoning. After removal of duplicates, full text publications of the remainder (n = 58) were retrieved and evaluated. Publications that did not provide data on non-fatal self-poisoning, and those that focused only on completed suicide were excluded (n = 42). 16 publications were retained after this full text screen, 6 more were added after the examination of bibliographies of the already retrieved publications and 4 were added after hand-searches of print issues of the Ceylon Medical Journal. Therefore in total 26 publications (representing 23 studies) were included in this review. Of the 23 studies included in the review, 3 studies were represented by 2 publications each, since these articles gave differing objectives and results. The 23 studies included in the review were of varying size and design. 18 were either cross sectional descriptive studies or retrospective studies based on medical case records (Table 1). One study included an intervention (brief intervention and contact following attempted self-harm) and was a randomized controlled trial [16]. In all except two studies, participants were inpatients admitted to hospital for medical treatment of acute self-poisoning. The remaining two studies were based in the community. When considered overall, 4 of the included studies were more robust in design, particularly with regards to methodology and study size [7,9,12,17]. Of these studies, one was a randomized nation-wide survey [12], two were large prospective studies (including >1000 participants each) [7,9], and the fourth comprehensively reviewed a described geographical area [17].

**Table 1:** Sri Lankan studies on attempted self-poisoning included in this review

**Randomized Controlled Trials (RCT):**				
**No**	**Study**	**Study Design**	**N**	**Outcomes examined**
1	Fleischmann et al. 2005 [13]	RCT of brief intervention following attempted suicide	1067	Demographic features, methods used and outcomes
2	Bertolote et al. 2010 [16]	Same study as above	TAU: 149 BIC: 151	Rate of repetition of attempted suicide at 18 months
**Case control:**				
**No**	**Study**	**Study Design**	**N**	**Outcomes examined**
3	Seneviratne et al. 1999 [37]	Case control	168 cases	Demographic features, psychiatric morbidity
4	Van Der Hoek et al. 2005 [14]	Case control	253 cases**of which 84% was intentional	Demographic features, types of poisons, risk factors
**Cross sectional descriptive:**				
**No**	**Study**	**Subjects**	**Number of subjects**	**Outcomes examined**
5	Fernando [35]	Subjects: patients hospitalized after poisoning	101	Demographic characteristics, poisons used
6	Chandrasena 1981 [29]	Subjects: patients hospitalized after poisoning	64	Demographic characteristics, poisons used, psychiatric morbidity
7	Jeyaratnam et al. 1987 [21]	Residents in the study area who had a history of hospital admission for poisoning + farmers in agricultural communities in 4 South Asian countries	94 (in Sri Lanka)**of which 36.2% was intentional	Types of pesticides used ingested, awareness among consumers of health hazards of pesticides
8	Hettiarachchi et al. 1989 [30]	Patients hospitalized due to self-poisoning in South Sri Lanka.	97	Demographic features, types of poisons, reasons for choice of poison and where obtained
9	Hettiarachchi et al. 1989 [33]	Same study as above	97	Intent, triggers, psychiatric morbidity
**Cross sectional descriptive continued:**				
**No**	**Study**	**Subjects**	**Number of subjects**	**Outcomes examined**
10	Eddleston et al. 2005 [7]	Patients hospitalized after self-poisoning, in a rural agricultural area, over one year	2189	Demographic characteristics, type of poisons ingested
11	Eddleston et al. 2006 [32]	Subjects: patients hospitalized after self-poisoning (opportunistic sample)	268	Reasons for choice of poison, outcome, expected outcome, premeditation
12	De Silva et al. 2008 [26]	Inpatients after self-poisoning (Colombo region)	191	Demographic characteristics, types of poisons ingested
13	Fahim et al. 2010 [19]	Inpatients after self poisoning (Polonnaruwa & Peradeniya regions)	816	Rate of previous self-harm
14	Dawson et al. 2010 [24]	Patients admitted to two rural hospitals after deliberate ingestion of a single pesticide, from 2002 to 2008.	9302	Demographic features, type of pesticide ingested
**Retrospective survey of medical records:**				
**No**	**Study**	**Type of records surveyed**	**Number of records**	**Outcomes examined**
15	Senewiratne et al. 1974 [18]	Records of all inpatients treated at Kandy Hospital for acute poisoning, in 1970 and 1971	472* *of which 82% was intentional	Rates of attempted poisoning, demographic features, types of poisons ingested
16	Dissanayake et al. 1974 [20]	Police records 1970–72, of the Police Post, General Hospital, Colombo region + Case notes of admissions for poisoning to Colombo Hospital 1970-72	270**of which 49% was intentional104 (non-random sample)	Demographic features (age and gender)
17	Jeyaratnam et al. 1982 [12]	Randomly selected hospital records of patients discharged with a diagnosis of pesticide poisoning, from hospitals throughout Sri Lanka	1000	Rates of poisoning, demographic features, types of poisons ingested
18	Senanayake et al. 1986 [36]	Hospital admissions for acute poisonings in hospitals in selected areas of Sri Lanka (Peradeniya, Colombo, Galle and Jaffna regions)	Peradeniya-179 Galle-100 Colombo- 101 Jaffna- 446	Demographic features, types of poisons ingested, associated illness
19	Hettiarachchi et al. 1989 [17]	Records of patients hospitalized due to non-fatal poisoning over a 1 year (1986–7) (South Sri Lanka)	669**of which 73% was intentional	Prevalence rates, demographic features, types of poisons, case fatality.
**Retrospective survey of medical records continued:**				
**No**	**Study**	**Type of records surveyed**	**Number of records**	**Outcomes examined**
20	Eddleston et al. 1999 [25]	Hospital records of patients treated for self ingestion of oleander plant (1995–96) + Assessment of inpatients after oleander ingestion	415 79	Demographic features, triggers for self-poisoning
21	De Silva et al. 2000 [34]	Hospital records of patients hospitalized due to parasuicide in Kandy, Peradeniya, Kurunegala and Matale regions during 1993–94.	5036* *of which >80% was intentional	Demographic features, type of poisons ingested
22	Van Der Hoek et al. 2006 [9]	Hospital records of patients hospitalized due to poisoning, in South Sri Lanka, from 1990–2002.	8110**of which 64% was intentional	Demographic features, rates of poisoning, type of poisons ingested
23	Manuel et al. 2008 [8]	Hospital records of patients admitted due to self-poisoning in rural south Sri Lanka + selected economic indices of that area	844	Rates of attempted poisoning, associations with socioeconomic indices
24	Senadheera et al. 2010 [27]	Hospital records of children & adolescents admitted to Hospital in South Sri Lanka (Karapitiya region), due to deliberate self-harm	827**of which 99% was due to attempted self-poisoning	Demographic features, types of substances ingested, change of substances ingested with time
**Qualitative:**				
**No**	**Study**	**Study Design**	**N**	**Outcomes examined**
25	Van Der Hoek et al. 1998 [23]	Mixed methods-Retrospective analysis of hospital records for information on occurrence of pesticide poisoning in the area + Qualitative interviews of families living in a village in a rural agricultural area	526**of which 68% was intentional	Quantitative: Socio-demographic features, types of pesticides ingested. Qualitative: Exploration of daily use, practices and storage regarding pesticides
26	Konradsen et al. 2006 [22]	Qualitative interviews with those who have attempted intentional self-poisoning, key workers in the area and focus group discussions with those from that community.	159	Exploration of factors and triggers associated with attempted self-poisoning (particularly sociological aspects).

### Rates of self-poisoning and repetition

Seven studies reported rates of self-poisoning (Tables 2 and 3). In all but one study [14], the data was derived from medical records of admissions to hospitals in the study area. The reported rates of non-fatal self-poisoning, either by pesticide ingestion, or by ingestion of any type of poison, increased in the three decades leading up to the turn of the century. In 1971–2 the reported rate of non-fatal self-poisoning (by any poison) was 26.2 per 100,000 (Kandy region)[18], whereas in 2002 it was 315 per 100,000 (Galle region) (Table 3) [8]. However except for one study [12], all reported self-poisoning rates were for specific regions in Sri Lanka rather than for the population of the country as a whole. The single island-wide survey conducted in 1981 [12] examined a random sample of clinical records of patients discharged during 1979 from all 10 general hospitals, and 5 of the 14 base hospitals, that provided inpatient care within the government health sector in Sri Lanka at that time. This study reported the rate of pesticide poisoning in Sri Lanka to be 79/100,000 population in 1979 (of which 73% was due to non-fatal self-poisoning), and also reported a clear variation in the rate of poisoning in different parts of the country, the highest rate being in the North and Northeast areas (Jaffna and Batticaloa regions) [12].

**Table 2:** Rates of self-poisoning (per 100,000 population) – pesticides only

**Authors**	**Period of study (Year)**	**Area studied**	**Rates of self-poisoning by ingestion of pesticides**
Jeyaratnam et al. 1982 [12]	1979	Sri Lanka (nationwide)	79/100,000**of which 73% was due to intentional self-poisoning
Van Der Hoek et al. 1998 [23]	1991-94	North-Central Province	260/100,000 per year to 290/100,000 per year**of which 68% was due to intentional self-poisoning
Van Der Hoek et al. 2005 [14]	1999	Uda Walawe region	163/100,000**of which 84% was due to intentional self-poisoning

**Table 3:** Rates for intentional self-poisoning (per 100,000 population) – for any type of substances including pesticides

**Authors**	**Period of study (Year)**	**Area studied**	**Rates of self-poisoning by ingestion of any type of substance**
Senewiratne et al. 1974 [18]	1971-2	Kandy region	26.2 /100,000**of which 82% due to intentional self-poisoning.
Hettiarachchi et al. 1989 [17]	1986-87	Galle region	54.7/100,000 (all due to intentional self-poisoning)
Van Der Hoek et al. 2006 [9]	1990-2002 Rate for year 2002	Southern Sri Lanka	318 per 100,000*of which 64% were due to intentional self-poisoning 350/100,000**of which 64% were due to intentional self-poisoning
Manuel et al. 2008 [8]	2002	Southern Sri Lanka	315/100,000 (all due to intentional self-poisoning).

The WHO SUPRE-MISS Study [16] prospectively examined repetition rates, where repetition was described as one or more suicide attempts following the baseline attempt of suicide (of which >90% was by self-poisoning), and reported that among the ‘treatment-as-usual’ group in Sri Lanka, 4% had repeated a suicide attempt by 18 months follow up. There were no other prospective studies on repetition rates following non-fatal self-poisoning. However two studies retrospectively examined rates of previous self harm (by any method) in those presenting to hospital with non-fatal self-poisoning, and reported previous self harm rates of 20% [14], and 8% [19].

### Substances used for self-poisoning

All except two studies [8,20] included in this review provided details on the types of poisons ingested by those who non-fatal self-poisoning. Six studies included only those who attempted poisoning by pesticide ingestion [12,14,21-24], and one study included only attempted poisoning by oleander ingestion [25]. The remaining 14 studies included in this review considered non-fatal self-poisoning in general. Among these 14 studies, in all but three [13,26,27], the most commonly ingested poison (by both sexes) was pesticides, with rates varying from 45.2% [28] to 77% [29]. Other ingested substances included pharmaceutical drugs, household chemicals – e.g., petroleum derivatives such as kerosene oil – and plant poisons, such as oleander. Non-fatal self-poisoning by pharmaceutical drug overdoses was reported in all 14 studies, but in most (11 studies) pharmaceutical overdoses occurred less frequently than pesticide ingestion. Of the three exceptions, two studies were conducted relatively recently (i.e., within the last 10 years) in urban Colombo – in one study pharmaceutical drug overdoses was the most common type of poison used by both sexes [26], and in the other it was the most common method of non-fatal self-poisoning in females [13]. The third such study examined non-fatal self-poisoning in adolescents (below 19 years), and here too pharmaceutical drug overdoses were the most common method used by females [27].

When considering patterns of substances ingested over time, studies published up to the turn of the century reported pesticides to be the most commonly ingested substance, irrespective of gender or region where the study was conducted (Table 4). Five studies included in this review were carried out after 2001, and of these, the studies conducted in urban areas reported an increasing frequency of medicinal overdoses over time (Table 4) [13,26].

**Table 4:** Types of poison ingested by males and females (studies listed in order of the year in which study was carried out, oldest first)

**Study**	**Year(s) and Place where study was conducted**	**First most commonly ingested poison type**		**Second most commonly ingested poison type**	
		**Among Males**	**Among Females**	**Among Males**	**Among Females**
Senewiratne et al. 1974 [18]	1970-1971 Kandy region	Pesticides	Pesticides	Medicinal overdose	Medicinal overdose
Chandrasena et al. 1981 [29]	1976 Kandy region	Pesticides	Pesticides	Medicinal overdose	Medicinal overdose
Fernando et al. 1977 [35]	1976 Colombo region	Pesticides (for both genders)		Medicinal overdose (for both genders)	
Senanayake et al.1986 [36]	1984 Peradeniya, Colombo, Galle, Jaffna regions	Pesticides	Pesticides	Medicinal overdose	Medicinal overdose
Hettiarachchi et al. 1989 [17]	1986-1987 Galle region	Pesticides	Pesticides	Kerosene	Kerosene
Hettiarachchi et al. 1989 [30]	1989 Galle region	Pesticides	Pesticides	Medicinal overdose	Medicinal overdose
De Silva 2000 [34]	1987-1991 Central Sri Lanka	Pesticides (for both genders)		-	-
Seneviratne et al. 1999 [37]	1996-1997 Ragama region	Pesticides (for both genders)		Medicinal overdose (for both genders)	
Van Der Hoek et al. 2006 [9]	1990-2002 Ratnapura, Monaragala, Hambanthota regions	Pesticides	Household products (mostly kerosene derivatives)	Pesticides	Household products (mostly kerosene derivatives)
Eddleston et al. 2005 [7]	2002-2003 Anuradhapura, Polonnaruwa regions	Pesticides	Pesticides	Oleander (plant)	Oleander (plant)
Fleischmann et al. 2005 [13]	2002- 2004 Colombo region	Pesticides	Medicinal overdose	Medicinal overdose	Pesticides
Senadheera et al. 2010 [27]	2001-2007 Galle region	Pesticides and other poisons (e.g. kerosene, household poisons)	Medicinal overdoses		
**Study**	**Year(s) and Place where study was conducted**	**First most commonly ingested poison type**		**Second most commonly ingested poison type**	
		**Among Males**	**Among Females**	**Among Males**	**Among Females**
Fahim et al. 2010 [19]	2005-2007 Peradeniya & Polonnaruwa regions	Pesticides	Oleander (plant)	Pesticides	Oleander (plant)
De Silva et al. 2008 [26]	2007 Colombo region	Medicinal overdose	Medicinal overdoses	Pesticides	Pesticides

Overall pesticide ingestion occurred more commonly among males than females, whereas medicinal overdoses, and the ingestion of plant poisons and petroleum derivatives was more common among females [9,13,17,25,30,31]. A study included in this review which examined trends of attempted self-poisoning among children and adolescents (aged 9–18 years), reported that overall a majority of girls took medicinal overdoses, whereas a majority of the boys ingested other poisons (such as washing powder, pesticides and kerosene oil) [27].

Three studies directly examined reasons for choice of poison in those who attempted poisoning, and in all three studies, the most commonly cited reason for choice was easy availability [26,30,32]. Of the three studies which reported on where the poison was obtained from, two studies reported that most persons (50-75%) obtained the poison from their own homes or gardens [30,32]. In both these studies pesticides were the most commonly ingested type of poison. However, the study in Colombo which reported medicinal overdose as the most self-poisoning was medicinal overdose reported that a majority obtained the drugs from pharmacies, purchased over the counter [26].

#### Socio-demographic factors

**Age distribution** Of the studies included in this review, 19 described the age distribution of those who carried out non-fatal self-poisoning. Four studies reported a peak age range of 15–24 years [26,29,33,34]. Another five studies described slightly broader, similar peak age ranges between 10–30 years [8,12,17,28,35], and most remaining studies described mean or median ages of below 30 years [7,9,13,14,20,24,25,32],[36,37].

**Gender distribution** All but one study [21] included in this review reported the gender distribution of those who carried out non-fatal self-poisoning. Most (16 studies) reported higher rates of non-fatal self-poisoning among males. In these studies the percentage of males ranged from 51.5% [30] to 72% [12]. Of the remaining studies, in one the ratio between the sexes was almost equal [19], and in five the rate was higher among females [14,25-27,37]. In three of the studies which reported a female preponderance of non-fatal self-poisoning, the type of poison most frequently ingested was medicinal overdoses and plant poisons rather than pesticides [25-27]. Gender distribution of non-fatal self-poisoning in urban areas, within the last 10 years, was reported in three studies only [13,26,27], and all three reported a higher prevalence of poisoning among females.

**Rural versus urban** No studies made direct comparisons between non-fatal self-poisoning in rural versus urban areas. Most studies (11 of the 23) were conducted in rural agricultural areas of the country. In the rurally-based studies, the rate of non-fatal self-poisoning was higher in males than females, and the most frequently ingested substance was pesticide. Of the remaining studies, five reported results from urban areas prior to 20 years ago (1985, 1976, 1971 & 1977), and the results of these studies were similar–pesticide ingestion was the most common mode of non-fatal self-poisoning [20,28,29,35,36], and rates of non-fatal self-poisoning were higher in males. In contrast, as noted above, two of the studies conducted in urban areas within the past 10 years (2007 & 2004) [13,26] among adults, reported a higher rate of non-fatal self-poisoning among females. In these latter two studies, pharmaceutical drug overdose was the most commonly used method by females in one study [13], with paracetamol being the most commonly ingested poison by both sexes in the second study [26]. Another study, conducted in both urban and rural areas during 2005–2006, reported that overall, the most commonly ingested substance was pesticides [19].

**Psychiatric morbidity** Nine of the twenty-three studies in this review reported psychiatric morbidity and alcohol use among those with non-fatal self-poisoning (Table 5) [14,22,25,29,33-37]. With regards to the method of psychiatric assessment, one study included assessment of each participant by a specialist psychiatrist [37], and another had used a questionnaire based on the Composite International Diagnostic Interview Short Form (CIDI-SF) [14]. The remaining studies were either interview based or based on perusal of medical records, and did not give details of the nature of the psychiatric assessment. Four studies reported rates of depression, and four studies investigated the presence of alcohol use disorders (Table 5). None of the studies investigated possible associations between non-fatal self-poisoning and other psychiatric conditions, such as anxiety disorders, impulse control disorders, or bipolar disorder. Of the four studies that examined for depression, one reported psychiatric illness in 13.4% of participants, of which depression was seen in 77% [33], one reported depression in 31 participants (18.5%) [37], whereas in contrast, one study found no significant association between self-poisoning and depression [14]. Three studies reported rates of alcoholism ranging from 2% to 10% [29,33,37]. Up to 50% of males with non-fatal self-poisoning were reported to be intoxicated at the time of the attempt [14,22,25].

**Table 5:** Rates of psychiatric illness and alcohol use in those who have attempted self-poisoning

**Study**	**Method of Psychiatric Assessment**	**Rates of psychiatric illness and alcohol use among study participants (and details where available)**
Fernando 1977 [35]	Participants interviewed as part of the study. Further details of psychiatric assessment not available.	• Psychiatric illness: 15.9% (this included schizophrenia, depression and mental retardation)
Chandrasena 1981 [29]	Participants interviewed as part of the study. Further details of psychiatric assessment not available.	• Psychiatric illness – 13%
		• Alcoholism – 2%
Senanayake et al. 1986 [36]	Information based on administrative records.	• Psychiatric illness - 5% (in Jaffna region) & 2.3% (in Peradeniya region)
		• Alcohol consumption at time of poisoning: 4% (in Peradeniya region)
Hettiarachchi et al. 1989 [33]	Participants interviewed as part of the study. Further details of psychiatric assessment not available.	• Psychiatric illness – 13.4% (Depression and schizophrenia present in equal numbers, in 77% of psychiatric illness)
		• Alcoholism - 7%
Seneviratne et al. 1999 [37]	A psychiatric assessment of each participant was conducted by a specialist psychiatrist.	• Depression – 18.5%
		• Schizophrenia – 1.2%
		• Alcoholism – 10.7%
Eddleston et al. 1999 [25]	Participants interviewed as part of the study. No formal psychiatric assessment.	• Alcohol intoxicated at the time of self-poisoning: 50% of male participants
De Silva et al. 2000 [34]	Data obtained from hospital records.	• Use of alcohol before/during self-poisoning: 6%
Van Der Hoek et al. 2005 [14]	A small subsample of the study population was assessed using a questionnaire based on the Composite Diagnostic Interview Short Form (CIDI-SF).	• Alcohol dependence is significantly associated with increased risk of self-poisoning.
		• No significant association between depression and self-poisoning (sub sample)
		• Alcohol intoxicated at time of self-poisoning: 36%
Konradsen et al. 2006 [22]	Based on interviews and focus group discussions.	• Life threatening illness or disability or mental illness– 8%
		• Alcohol intoxicated at time of self-poisoning: 32% (all males)

### Premeditation, triggering factors and suicidal intent

Four (of the twenty-three) studies reported the duration of premeditation prior to the self-poisoning act [25,26,32,33]. These studies found little premeditation. In one study 74% of the self-poisoning acts occurred within 4 hours of making the decision to ingest poison [33], and in another, more than half of the participants reported ingesting the poison within 30 minutes of an interpersonal conflict (argument) [25]. In addition, a study from urban Colombo reported that of those who purchased medication for the purpose of overdose, about 80% made the purchase within one hour of ingestion [26].

Six studies identified triggers that precipitated the non-fatal self-poisoning act [22,25,29,32,33,35]. Of these, five studies reported that the commonest precipitant (in >50% of those who self-poisoned) was interpersonal conflict. Common examples included domestic disputes and romantic relationship problems, leading to arguments with family members [33]. Konradsen et al. [22] described alcohol misuse as a significant factor in non-fatal self-poisoning among Sri Lankan males. This study also described ways in which alcohol misuse (among males) led to domestic violence and interpersonal conflict within the home, thereby indirectly contributing to increased risk of self-poisoning in the misuser as well as his wife and children.

Intent to die (at the time of the attempt) was directly reported in only two studies. One of these studies which reported on participants after non-fatal self-poisoning by ingestion of any substance, found that 55.7% wished to die (and 27% retained that wish after surviving the act) [33]. The other study [25] which examined only non-fatal self-poisoning by ingestion of oleander seed (a plant poison) reported that most who attempted did not want to die.

## Discussion

### Rates, and types of poisons used

Despite the reported drop in completed suicide rates in Sri Lanka after 1995 [1], the rates of non-fatal self-poisoning in this country have increased in the three decades leading up to the turn of this century [8,9,12,18,19] (Tables 2 and 3), and this trend is seen both for non-fatal self-poisoning by pesticide ingestion, as well as for poisoning by ingestion of any other substance. This is supported by the more recent findings of De Silva et al. [38], who reported that the rate of hospital admissions for any type of poisoning in Sri Lanka has increased from 204.8 admissions per 100,000 population in 1995, to 321.2 per 100,000 in 2007 – this despite a clear drop in the rate of completed suicides during the same period. The reported non-fatal self-poisoning rates for Sri Lanka are also high when compared with rates of self-poisoning in other developing countries, including Turkey (145 per 100,000) [39] and Suriname, South America (284 per 100,000) [40]. In Europe, the WHO/Euro multicentre study reported the highest average male-standardized attempted suicide rate to be 314/100,000 in Helsinki, Finland [10], a rate not dissimilar to the rate of non-fatal self-poisoning reported for males in Galle, Sri Lanka in 2002 (330/100,000) [8] (Table 3).

Direct country comparisons between rates of non-fatal self-poisoning require carefully interpretation. First, in the Sri Lankan studies, most rates are based on regional samples, and are not necessarily generalizable to the entire country. Data from 1979 showed hospital admission rates for pesticide poisoning to be highest in the North of the country [12]. Second, an important confounder is that most of the available rates are derived from studies based on hospital admissions. Thus, factors influencing the rates of admission to hospital following non-fatal self-poisoning – such as improvement in transport services and increased numbers of peripheral hospitals – could impact on study findings over time. The toxicity of ingested substances may also influence hospital admission rates – prior to the restriction of the sale of toxic pesticides such as WHO Class I toxicity pesticides in Sri Lanka [1], many who ingested pesticides may have died prior to admission to hospital.

As expected, when considered overall, the most commonly ingested substance with regards to non-fatal self-poisoning in Sri Lanka was pesticides (Table 4), similar to neighbouring countries such as India [41]. However, two studies carried out after 2001 in urban areas of the country, report medicinal overdoses to be the most common substance used, for both genders in one study [26], and among females in the other study [14] (Table 4). Interestingly, two studies included in this review reported on changes in types of poisons ingested over time [9,27], of which the more recent study by Senadheera et al. [27] of young people aged less than 19 years in urban Galle reported a dramatic increase of medicinal overdoses from 2001 to 2007. This included a five-fold increase of paracetamol overdose from 2001 to 2007. The findings of this review suggest that there has been an increasing rate of pharmaceutical drug overdoses rather than pesticide self-poisoning during the last decade in more urbanized areas of the country. The recent review by De Silva et al. reported similar findings for Sri Lanka, i.e., increased hospital admissions for poisoning by medicinal and biological substances, and decreased admissions due to pesticide ingestion since 2003 [38]. Interestingly, a recent study published in 2012 reports that while the highest rates of non-fatal self-poisoning are seen in agricultural areas (e.g., Anuradhapura, Polonnaruwa, Hambanthota), even in these areas there is a rapid increase in self-poisoning with medicinal overdoses and other biological substances [42]. The reason for these changes has not been directly investigated. However, given that the most common reason for choice of poison is easy availability [26,30,32], the finding may reflect the increasing accessibility of medicines relative to pesticides, particularly with urbanization. This trend has important implications, for future health policy in Sri Lanka. It suggests that policy makers should consider introducing preventive strategies such as restriction of the quantity of paracetamol available as a single purchase, in order to reduce risk of medical complications associated with self-poisoning due to overdoses [43].

### Demographic factors of those who attempt self-poisoning

There are both similarities and differences in the patterns of demographic characteristics among non-fatal self-poisoning in Sri Lanka and other countries. In Sri Lanka non-fatal self-poisoning is seen predominantly among young adults aged between 15 and 30 years. This is similar to self-poisoning patterns described elsewhere, in both the developed and developing world [6,10,40,44-47].

By contrast, the gender distribution of non-fatal self-poisoning in Sri Lanka differs from that in Western countries. In the majority (14) of the studies included in the review, the rate of non-fatal self-poisoning in Sri Lanka was greater in males than females. This is the reverse of the findings from Western countries [10,44,46]. For instance, in the WHO/EURO multicentre para-suicide study, in all except one centre (Helsinki) the suicide attempt rate (a majority by non-fatal self-poisoning) was higher for females, the average male: female ratio being 1:1.5 [10]. However, gender distribution patterns similar to Sri Lanka have been reported from elsewhere in Asia [47-49].

The reason for the higher rate of non-fatal self-poisoning among males in Sri Lanka is not clear. One possible influencing factor is the pattern of alcohol consumption in this country. Sri Lanka is reported to have a high level of alcohol consumption, as indicated by an increase in alcohol related health problems in recent years [50], and culturally, alcohol use is much more common among men compared to women in this country. Alcohol use disorders are known to be associated with suicidal behaviours [51], and in keeping with this, Sri Lankan studies have reported that up to 50% of men were under the influence of alcohol at the time of the self-poisoning act [14,22,32].

When examining the gender ratios according to region, Sri Lankan evidence indicates that the rates of non-fatal self-poisoning in males are higher than females primarily in rural agricultural areas of the country. Again the reasons for this are unclear. One hypothesis is that men living and working in rural agricultural areas have easy access to pesticides stored in the fields or gardens. Previous work has also suggested that continued exposure to pesticides itself may increase suicidal ideation [52], but this remains an area for future research.

In contrast to the findings from the rural agricultural areas, two studies conducted relatively recently in urban areas of Sri Lanka [13,26] have reported a higher rate of non-fatal self-poisoning for females, and in one study the most common substance used for non-fatal self-poisoning was pharmaceutical drug overdose rather than pesticide ingestion [26]. This appears to signify a trend that has been emerging in the last decade, of an increasing ratio of non-fatal self-poisoning among females compared to males in urban areas, paralleled by an increase of pharmaceutical drug overdoses rather than pesticide self-poisoning in these areas. Other developing countries too have shown similar gender differences of non-fatal self-poisoning in rural compared to urban areas [45,53]. For example, a study undertaken in Hanoi, Vietnam [45] reported a higher female-to-male ratio of non-fatal self-poisoning in urban areas where medicinal overdose is more common, compared to rural areas where pesticides ingestion is more common. Notably, non-fatal self-poisoning by medication is higher among females than males in Western countries [10].

### Premeditation and precipitating factors

The most common associated or precipitating trigger for non-fatal self-poisoning in Sri Lanka was interpersonal conflict, most commonly with a close family member. In contrast, reportedly less than 5% of self poisoning was precipitated by financial difficulties [22] similarly Hanwella et al. [42] found no clear association between poverty rates and non-fatal self-poisoning rates among the different districts in Sri Lanka. Interpersonal conflict has been reported to be the acute trigger associated with more than 60% of self poisonings in Pakistan and India [6,54] as well. Previous authors have suggested that self-poisoning in Sri Lanka may be considered by some as an acceptable way of coping with stress and conflict [55,56], and that those who self poison often know of others who have done the same [32]. Marecek et al. have suggested that increasing non-fatal self-poisoning in women in particular maybe a reflection of the clash between the emergent expectations of young women about their education, relationship and employment, and the more traditional ideals of feminine behavior held by their elders, occurring against a background of development and modernization [57]. The collectivistic rather than individualistic nature of society in this country [58], the hierarchical framework where overt confrontation is discouraged, and the strong sense of shame which is associated with loss of face [59], may all be causes of self-poisoning in response to interpersonal conflict.

The available evidence suggests that non-fatal self-poisoning in Sri Lanka is associated with brief premeditation. The two studies in this review, which examined suicidal intent associated with the non-fatal self-poisoning, reported conflicting result [25,33]. One study which examined self-poisoning by ingestion of any poison reported suicidal intent in up to 55.7% of those who attempted [33], whereas the other study which examined self-poisoning by oleander seed ingestion reported that most did not wish to die [25]. One possible explanation for the discrepancy in the findings may be the differences in substances ingested in the two studies concerned. Oleander seed is a plant poison available in the garden, and may be more associated with impulsive attempts of self-poisoning. Differences in the methods used in the two studies for assessing suicidal intent may also have influenced the findings.

### Risk of repetition and psychiatric morbidity

Prospective follow up data from South Asia on suicide attempts following an index suicide attempt is limited. The only prospective study of non-fatal self-poisoning in Sri Lanka, found that the repetition rate of 4% following an index episode of non-fatal self poisoning in Sri Lanka, which is comparable to repetition rates reported for Yungcheng in China and Chennai, India [16]. This is consistent with the findings from a prospective study of 140 persons admitted to a Sri Lankan hospital following attempted suicide by *any* methods which found no subsequent suicide attempts during the follow up two year period- although it is a limitation of the study that only 61% of the participants could be reviewed at the end of the two years [34]. In contrast to these low rates in Sri Lanka, prospective follow up studies conducted in the West have reported repetition rates of 15% and above [10,60,61]. The reason for this difference is not clear. One possibility might be that results in Sri Lanka are confounded by a high case fatality - those who would be potential repeaters in the West may be dying in the first attempt in Sri Lanka. The paucity of prospective studies in Sri Lanka is another limitation, and precluded an exploration of the factors associated with repetition of self-poisoning. However, evidence from retrospective studies highlights the importance of understanding such factors. Whereas repetition rates reported in those following *non-fatal* self-poisoning is low, the rate of prior suicidal behaviours in those who have *completed* suicide (mostly by poisoning) in Sri Lanka is 26% [62,63]. Identifying this high risk group from among those who attempt suicide is an important challenge for future research.

The evidence available suggests that rates for depression in those who self-poison in Sri Lanka are low [14,33], in contrast to those in the west, where rates of over 40% have been reported [44]. The low levels of depression reported among those who attempted self-poisoning in Sri Lanka may reflect true levels; alternatively the presence of depression may have been overlooked, and evidence from India suggests that the risk factors for suicide in Asia are similar to those described elsewhere in the world [64]. It is difficult to differentiate these possibilities given that only four studies have reported rates of depression in this population, and neither provided detailed descriptions of the methods employed to assess psychiatric morbidity. Furthermore depression in men may have been concealed by reported alcohol dependency or alcohol use disorder [65].

Alcohol use disorders have been reported to be associated with self-poisoning behavior in Sri Lanka [14,37], which is similar to findings internationally [15]. There is no evidence regarding the rates of impulse control disorders or anxiety disorders in those who attempt self-poisoning in Sri Lanka, although Nock et al. [15] have reported that impulse control disorders and anxiety to be predictive of suicidal behavior in the developing world. There is also no evidence available regarding non-fatal self-poisoning in Sri Lanka and factors such as chronic stressors, hopelessness, and family history of suicide. Family history of suicide and chronic long term stressors have been shown to increase the risk of completed suicide in other Asian countries such as China, with the presence of multiple risk factors are associated with increased risk [66-68].

### Implications for future interventions

Several key aspects emerge from this review. First, the findings from several studies suggest that non-fatal self-poisoning in Sri Lanka is associated with brief premeditation in the context of acute interpersonal stress, albeit that no study investigated concurrent long term stressors or other vulnerability factors which might have increased the risk of non-fatal self-poisoning in the context of interpersonal stress. This finding suggests that one potential area of intervention to reduce rates of non-fatal self-poisoning in Sri Lanka is at a primary preventive level, through community programs aimed at developing interpersonal skills and skills for coping with interpersonal stress. Given the young age group most at risk and the apparently low rate of repetition, a prevention approach that targets older teenagers in schools and young adults in the community is indicated. Similar preventive strategies have been suggested previously for Sri Lanka [56] as well as other Asian countries such as Vietnam [45]. Particular care would need to be taken to tailor the intervention in a culturally appropriate and acceptable manner, and further research would be needed to determine the feasibility and effectiveness of such methods.

Second, alcohol use disorders are known to be associated with suicidal behaviours [51], and Sri Lanka is no exception, especially with respect to self-poisoning among males. Community and national level strategies to reduce alcohol misuse is an essential, albeit challenging, area of intervention to reduce rates of attempted self-poisoning. Culturally compatible interventions, such as the community based educational program to reduce alcohol misuse reported by Siriwardhana et al. [69], should be considered in this regard.

Third, there is a paucity of information available regarding psychiatric morbidity associated with non-fatal self-poisoning in Sri Lanka, although studies from countries such as China have reported that high-intent suicide attempts are associated with depression and chronic stress [70]. Prospective follow up studies of psychiatric morbidity, family history, and chronic stressors of non-fatal self-poisoning in Sri Lanka, are needed to inform future interventions and to assist in identifying risk factors for future repetition. Those presenting to services following non-fatal self-poisoning could then be screened for factors associated with a higher risk of repetition, and persons thus identified referred for further specialized assessment and care. This would be a potentially cost effective intervention at a secondary prevention level.

Finally, it should be noted that the factors associated with non-fatal self-poisoning are complex and multiple, and an integrated, multifactorial approach towards reducing the rate of non-fatal self-poisoning is likely to be more effective than focusing on single risk factors. Phillips et al. [68] have suggested a similar multifactorial approach towards suicide prevention in China. Similarly, a broad based integrated approach, which combines multiple components, such as community based programs to develop interpersonal skills in young people, community interventions to reduce alcohol misuse, and the identification and specific management of those who are at higher risk of repetition of attempted suicide, are likely to be the most effective in reducing self-poisoning rates in Sri Lanka in the long term. Such interventions must also be responsive to the rapidly evolving role shifts and increasing urbanization occurring in Sri Lanka. Safe storage of pesticides has already been suggested as a method of reducing the burden of non-fatal self-poisoning in this country [71]. Furthermore, the emerging shift from pesticide ingestion to medicinal overdoses indicates that it is also timely to consider restricting over-the-counter sale of pharmaceutical items such as paracetamol, as has been suggested for the West [72]. Similar integrated preventive measures have been proposed for other Asian countries [73,74], and further research is required to explore the effectiveness of such approaches.

#### Limitations

A primary limitation of this review is that it is based on publications in indexed peer reviewed journals, and thus findings maybe limited by publication bias. Studies and abstracts presented at conferences, in non-indexed journals, as well as other unpublished literature were not incorporated. As far as possible, attempts were made to ensure the search was as inclusive as possible, by searching multiple electronic databases, and by examining bibliographies of already selected articles for any further publications of relevance. A hand search was also conducted of the archives of the Ceylon Medical Journal, which is the oldest indexed medical journal in Sri Lanka. The search was conducted in the English language, but since the language used for scientific and medical publications and conferences in Sri Lanka is English, a language bias is unlikely.

Other limitations include the fact that the search included publications up to 31^st^ October 2011 only, and 5 potentially eligible articles published since October 2011 could not be included. The mixed nature of the study types, which makes comparison between studies challenging, and the limited details available on certain aspects of self-poisoning in Sri Lanka such as psychiatric morbidity are also limitations. The variation in the year in which the studies were conducted, which ranged from 1974 to 2011, may also have influenced relevance of findings to current preventive strategies. Many of the findings reported in this review are based on cross-sectional data, which provide limited information about rates of change and factors associated with attempted self-poisoning. Furthermore, as mentioned previously, the aim of this review was to examine factors associated with non-fatal self-poisoning in Sri Lanka. However, due to the nature and paucity of the studies available, the review included studies which focus on intentional self-poisoning where the outcome (i.e., survival or death) was not differentiated. This is a limitation of the review. Another possible limitation is that during data extraction, equal weight was given to all studies, irrespective of study quality. However the overall findings were largely supported when higher quality studies were examined individually [7,9,12,17].

## Conclusion

The rates of non-fatal self-poisoning in Sri Lanka have increased in recent years, despite a clear decrease in the rate of completed suicides since 1995. With respect to features such as gender ratio, methods used and rates of self-poisoning, Sri Lanka shares similarities with other Asian countries rather than the West. However intriguing recent evidence indicates that medicinal overdoses are becoming more common, and this has been paralleled by a gender shift towards increased female self-poisoning in urban areas - an apparent change towards non-fatal self-poisoning patterns as seen in the West.

Non-fatal self-poisoning in Sri Lanka is reported to be associated with interpersonal conflict, with short premeditation, and also to be associated with alcohol misuse among males. There is a dearth of information about potential associations with other factors such as psychiatric morbidity and chronic stressors. Reduction of attempted suicide rates needs to be a national priority, and available evidence suggests the need for integrated intervention strategies which encompass several broad aspects, namely community based development of interpersonal skills among young people, community based programs to reduce alcohol misuse, plus screening for and specific management of those at high risk of repetition following non-fatal self-poisoning. This remains a challenging area, which requires further research to explore the effectiveness of such an approach, and findings for Sri Lanka may have implications for similar intervention programs in other South Asian countries as well.

## Competing interests

No financial or non-financial competing interests.

## Authors’ contributions

Authors (TR), (HC) and (KMG) were involved in the conceptualization and planning of the study. TR did the preliminary literature searches, which was rechecked and modified by HC and KMG. All three authors were actively involved in writing and revising the article, and all three have seen and approved the final version submitted for publication. All authors read and approved the final manuscript.

## Pre-publication history

The pre-publication history for this paper can be accessed here:

http://www.biomedcentral.com/1471-2458/13/331/prepub
